# Case report: Novel TBX5-related pathogenic mechanism of Holt–Oram syndrome

**DOI:** 10.3389/fgene.2023.1063202

**Published:** 2023-03-01

**Authors:** Yuheng Lang, Yue Zheng, Bingcai Qi, Weifeng Zheng, Chengxiu Zhao, Hu Zhai, Gang Wang, Zhiqiang Luo, Tong Li

**Affiliations:** ^1^ Department of Heart Center, Tianjin Third Central Hospital, Tianjin, China; ^2^ School of Medicine, Nankai University, Tianjin, China; ^3^ Tianjin Key Laboratory of Extracorporeal Life Support for Critical Diseases, Tianjin, China; ^4^ Artificial Cell Engineering Technology Research Center, Tianjin, China; ^5^ Tianjin Institute of Hepatobiliary Disease, Tianjin, China; ^6^ Department of Anesthesiology, Handan First Hospital, Handan, China

**Keywords:** Holt–Oram syndrome, Tbx5, ChIP, case report, SNP

## Abstract

**Introduction:** Holt–Oram syndrome (HOS) is a rare genetic disorder characterized by upper limb abnormalities, congenital heart defects, and/or conduction abnormalities. Sequence alteration of T-box transcription factor 5 (TBX5) is correlated with the incidence of HOS.

**Case description:** We present the case of a 24-year-old female with upper limb alterations (congenital dysplasia in the wrist and elbow joints) and an anomalous left main trunk arising from the right coronary sinus. The patient inherited a base T (reference C) at rs883079 from her mother and base C (reference T) at rs10850326 from her father, both of which belong to the 3′-untranslated region (UTR) of the TBX5 gene; no alterations in TBX5 expression or single-nucleotide polymorphisms (SNPs) in other exon areas were found. We explored the effects of TBX5 on cardiomyocytes using the HL-1 cell line and TBX5-knockdown cells.

**Discussion:** Quantitative polymerase chain reaction analysis demonstrated that TEKT2, TEKT4, and SPTB expression decreased after TBX5 knockdown, while chromatin immunoprecipitation analysis further revealed that TBX5 binds to the TEKT2, TEKT4, and SPTB promoter regions to promote gene transcription. Our findings support a novel TBX5-related pathogenic mechanism in HOS.

## 1 Introduction

Holt–Oram syndrome (HOS; MIM 142900) is a rare genetic disorder characterized by upper limb abnormalities, congenital heart defects, and/or conduction abnormalities (Barisic et al., 2014). The uncommon subtypes of HOS are Tabatznik syndrome (type II; arrhythmias and brachytelephalangy), a Spanish variant (type III; arrhythmia and brachydactyly type C), and a potential Slovenian type (type IV; arrhythmia, dilated cardiomyopathy, and brachydactyly) (Zaragoza et al., 2017; Spiridon et al., 2018).

HOS is caused by a mutation in the T-box transcription factor 5 (TBX5) gene (located on chromosome 12q24.1), a protein-coding gene consisting of nine coding exons and belonging to the T-box domain (Spiridon et al., 2018). The Nkx2.5/GATA4/TBX5 axis controls heart and limb development, and the abnormal expression of TBX5 induces abnormalities by altering the expression of some critical genes, such as ANF and KLF13 (Darwich et al., 2017; Russell et al., 2018). Mutations in TBX5 were identified in more than 70% of HOS cases. Pathogenic TBX5 variants include missense or non-sense substitutions, large deletions of multiple exons, frameshift mutations, and intragenic duplications (Spiridon et al., 2018).

Because of its rare occurrence and consistent phenotypes and symptoms, only a few studies have been conducted on HOS development and little is known of the functions of its molecular components. In this study, we aimed to describe a novel TBX5-related pathogenic mechanism of HOS in which there was no mutation in TBX5 or single-nucleotide polymorphisms (SNPs) in other exon areas except the 3′-untranslated region (UTR). Our study findings could promote the development of more effective prognostic and treatment tools and enable early identification of individuals at risk of transferring the disease.

## 2 Case description

In December 2021, a 24-year-old female was admitted to the hospital because of intermittent chest tightness and suffocation for 6 years, followed by 3 months with no medical history. The results of the medical examination were as follows: temperature 36.8°C, pulse 72 times/min, respiratory rate 16 times/min, and blood pressure 113/76 mmHg. More results included clear, stable breathing, coarse breath sounds in both lungs, no rale or wet rale, no uplift in the precardiac area, strong heart sound, consistent heart rhythm (HR 72 beats/min), no murmur in the auscultation area of each valve, flat and soft abdomen, no tenderness, internal rotation malformation of both upper limbs, no external rotation, and no edema in either of the lower limbs.

**FIGURE 1:**
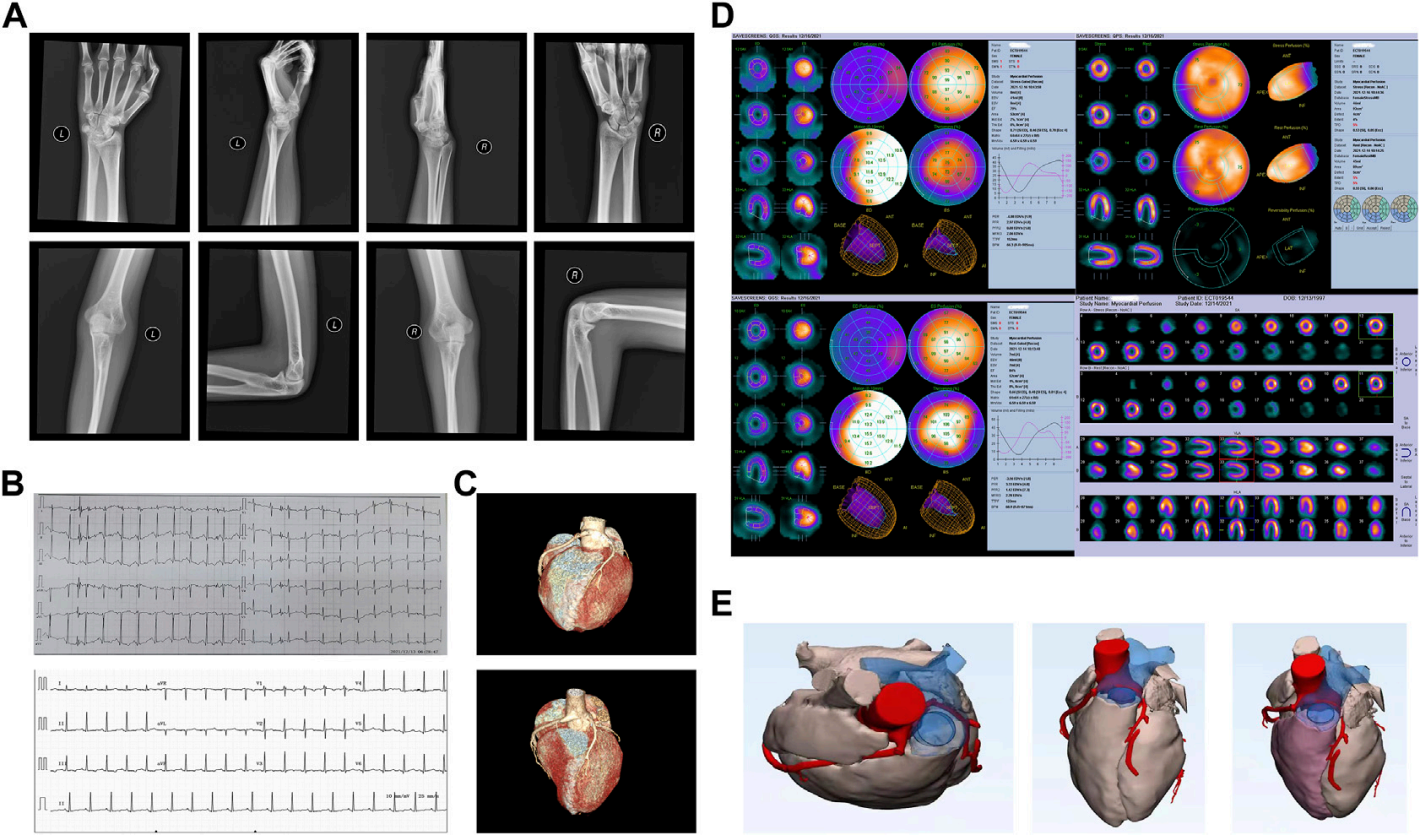
Patient’s clinical characteristics. **(A)** Bilateral elbow wrist joint examination, demonstrating a partial fusion of bones at the proximal end of both the ulna and radius, widening of the radial ulnar joint at the distal end, and poor alignment. **(B)** Electrocardiography before (top) and after surgery (bottom). **(C)** Computed tomography angiography of the cardiac structure before (top) and after surgery (bottom). **(D)** Resting and adenosine load-gated myocardial perfusion imaging demonstrated that there was no typical reversible abnormal perfusion in the left ventricular myocardium, and the left ventricular cardiac function index was normal. **(E)** Three-dimensionally printed heart model used to determine the surgical approach.

A bilateral elbow wrist joint examination demonstrated the partial fusion of the bones at the proximal end of both the ulna and radius, widening of the radial ulnar joint at the distal end, and poor alignment, suggesting congenital dysplasia in the wrist and elbow joints (Figure 1A). Cardiac color ultrasonography showed no abnormalities in the cardiac structure or function in the resting state (left ventricular ejection fraction = 65%). Outpatient coronary computed tomography angiography and angiography after admission revealed that her left coronary artery originated from the right coronary sinus and the slender left coronary artery was contorted between the aorta and the pulmonary artery (Figures 1B, C). In the cardiac systolic movement, severe stenosis appeared in the left coronary artery opening; however, no obvious stenosis was observed in the right coronary artery, left descending artery, or left circumflex artery. Resting and adenosine load-gated myocardial perfusion imaging revealed no typical reversible abnormal perfusion in the left ventricular myocardium and showed that the left ventricular cardiac function index was normal (Figure 1D).

The transient heart rate increased and chest tightness appeared after admission. An electrocardiogram examination suggested a high sinus heart rate, HR 123 beats/min, and ST segment depression. A three-dimensional printed model was used to determine the appropriate surgical approach (Figure 1E). Midline thoracotomy was performed by cardiopulmonary bypass. We found that the left main trunk was deformed between the ascending aorta and the main pulmonary artery and arose from the right coronary sinus; therefore, correction surgery for coronary artery malformation was performed to reconstruct the left main trunk. On the first day after surgery, the patient had a heart rate of 130 beats/min and no other symptoms, such as chest tightness, appeared. A coronary computed tomography angiographic examination was performed again after surgery, which demonstrated that the left main trunk had returned to a normal shape.

To investigate the genetic etiology, we performed mutation and SNP analyses of the patient’s family using SAMtools and ANNOVAR (Novogene, Beijing, China). The screened mutations and SNPs were correlated using ClinVar (https://ncbi.nlm.nih.gov/clinvar/), which indicated that the patient inherited a base T (reference C) at dbSNP ID rs883079 from her mother and base C (reference T) at dbSNP ID rs10850326 from her father, both of which belong to the 3′-UTR of the TBX5 gene (Table 1). However, no alterations in TBX5 expression or SNPs in other exons were found. The patient also inherited a SALL4 SNP in the exon area from both her father and mother. We identified 578 exons, 498 3′-UTRs, and 346 5′-UTR mutations in the patient, including the female urogenital genes PKD1, SON, and BMPR1B, as well as the reproduction-related genes TEKT2, TEKT4, COL11A1, ANKRD11, LEMD3, and SPTB (Supplementary Materials: Raw Data).

**TABLE 1:** TBX5 SNPs among the Holt–Oram syndrome patient, her father, and her mother.

	CHROM	POS	dbSNP ID	REF	ALT	Func	Subcellular_location
Patient	12	114793240	rs883079	C	T	UTR3	Nucleus but not nucleoli
Patient	12	114793297	rs10850326	T	C	UTR3	Nucleus but not nucleoli
Patient	12	114832510	rs2236017	C	A	Intronic	Nucleus but not nucleoli
Father	12	114793297	rs10850326	T	C	UTR3	Nucleus but not nucleoli
Father	12	114794057	rs2113433	T	G	Intronic	Nucleus but not nucleoli
Father	12	114823098	rs77090182	C	T	Intronic	Nucleus but not nucleoli
Father	12	114832510	rs2236017	C	A	Intronic	Nucleus but not nucleoli
Mother	12	114793240	rs883079	C	T	UTR3	Nucleus but not nucleoli
Mother	12	114794057	rs2113433	T	G	Intronic	Nucleus but not nucleoli
Mother	12	114823089	rs17731453	G	A	Intronic	Nucleus but not nucleoli
Mother	12	114823098	rs77090182	C	T	Intronic	Nucleus but not nucleoli
Mother	12	114832510	rs2236017	C	A	Intronic	Nucleus but not nucleoli
Mother	12	114832727	rs181002410	A	G	Intronic	Nucleus but not nucleoli

Red, the TBX5 SNP inherited from her mother; blue, the TBX5 SNP inherited from her father.

**FIGURE 2:**
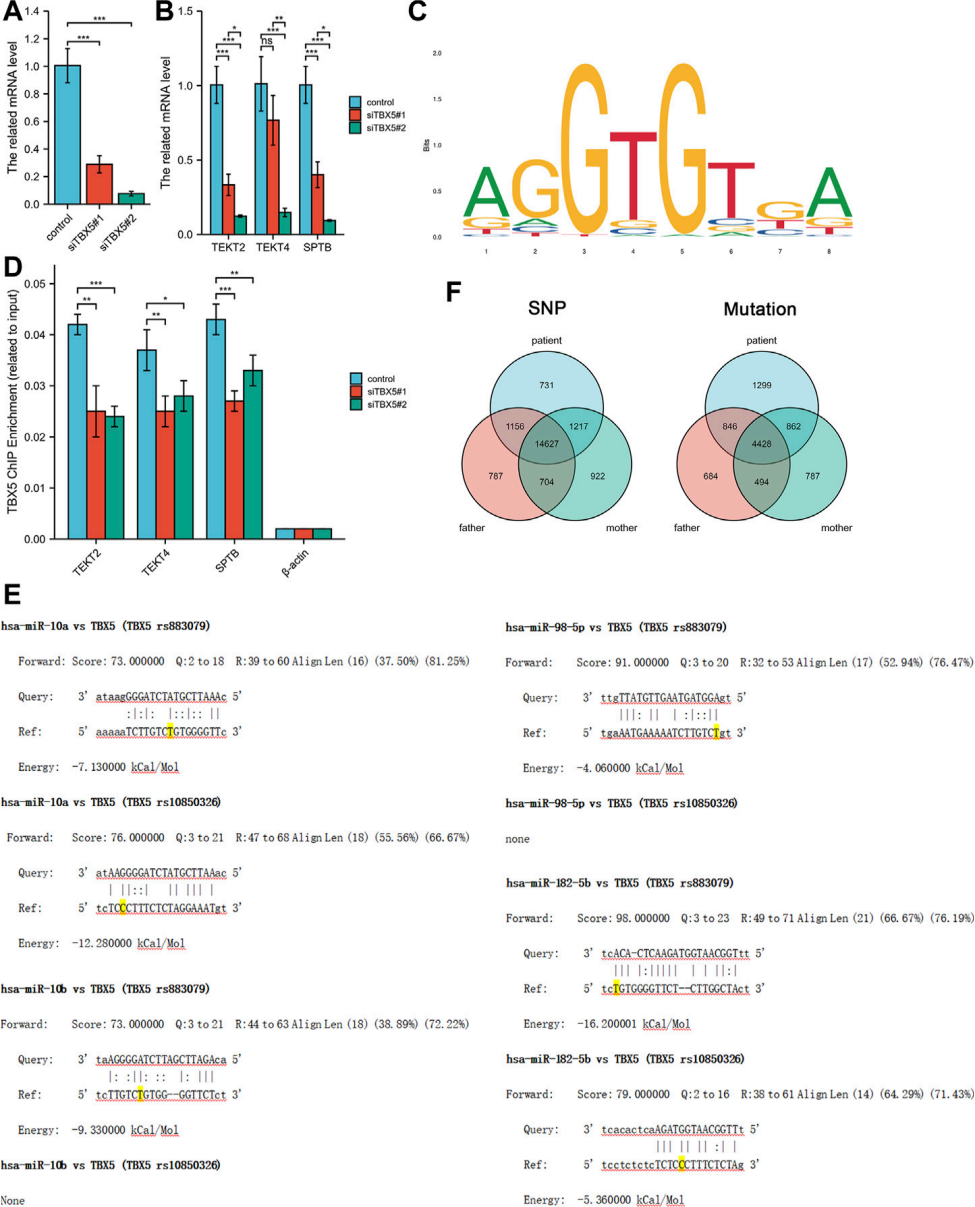
Effects of TBX5 expression on cardiomyocytes. **(A)** TBX5 expression after TBX5 knockdown in the HL-1 cell line by transfection of small interfering RNAs. **(B)** Expression of TEKT2, TEKT4, and SPTB decreased after TBX5 knockdown, indicating that they are downstream of TBX5. **(C)** Chromatin immunoprecipitation analysis showed that binding of TEKT2, TEKT4, and SPTB DNA fragments to TBX5 decreased after TBX5 knockdown, demonstrating that TBX5 promoted TEKT2, TEKT4, and SPTB transcription by binding to the promoters. β-Actin was used as the internal control. **(D)** Motif of binding sites between TBX5 protein and the promoters of the DNA fragments. **(E)** Sequence alignment between TBX5 3′-UTRs, including rs883079 and rs10850326, and microRNAs (miRNA), including hsa-miR-98-5p, hsa-miR-10a, hsa-miR-10b, and hsa-miR-182-5p. The bases on the rs883079 and rs10850326 are labeled yellow. **(F)** Venn diagram of single-nucleotide polymorphisms and mutations in the HOS patient’s family determined using whole-exome sequencing. *p < 0.05, **p < 0.01, and ***p < 0.001; ns, not significant.

We explored the effects of TBX5 on the HL-1 cell line with TBX5 knockdown *via* transfection of small interfering RNAs (Ubigene, Guangzhou, China; Figure 2A). Quantitative polymerase chain reaction analysis demonstrated that the expression of TEKT2, TEKT4, and SPTB decreased after TBX5 knockdown (Figure 2B), and chromatin immunoprecipitation analysis further revealed that TBX5 binds to TEKT2, TEKT4, and SPTB promoter regions to promote gene transcription (Figures 2C, D; Table 2). We performed sequence alignment between the TBX5 3′-UTR regions, including rs883079 and rs10850326, and microRNAs (miRNA), including hsa-miR-98-5p, hsa-miR-10a, hsa-miR-10b, and hsa-miR-182-5p. All four miRNAs could bind to the TBX5 3′-UTR that included rs883079, while only hsa-miR-10a and hsa-miR-182-5p could bind to the region that included rs10850326, demonstrating that the increased binding of miRNAs and TBX5 3′-UTR in HOS further decreased TBX5 expression (Figure 2E). These SNPs and familial mutations may be associated with the differential expression of TBX5 (Figure 2F).

**TABLE 2:** Specific primers used for quantitative real-time PCR.

Name	F	R
TEKT2	ATG​GCG​ACA​CTA​AGT​TTC​AAG​C	TGG​CGG​ATT​TGG​TGG​GAA​G
TEKT4	GGC​CCC​ACA​GTC​AAT​AGA​TGT	GGG​CAT​AAG​AGT​TCT​GAA​ACC​A
SPTB	GCT​CCC​ACG​ACA​TTG​TAG​ATG	GTG​TTT​CAC​GAC​CTT​CCT​GAG
TEKT2 promoter	CGA​GAA​CAT​GGA​AAA​CGG​GTG	CTC​TTA​AAA​TGT​GTT​TCC​TGC
TEKT4 promoter	TGA​GAC​AGA​GGA​GCA​GGG​CAA	TTG​TTG​GAA​ACA​GAA​TCG​ACA
SPTB promoter	GCT​GGT​AGC​AAG​GCT​GGG​ACA	CTG​TTT​TTG​AGC​AGG​CCA​GTC
β-Actin	GGC​TGT​ATT​CCC​CTC​CAT​CG	CCA​GTT​GGT​AAC​AAT​GCC​ATG​T

## 3 Discussion

TBX5 is located in the long arm of chromosome 12 (12q24.1) and it plays a fundamental role in tissue and organ formation during embryonic development and in maintaining the normal development of the upper limbs and heart (structure and electric system) through the activation of different genes (Stennard and Harvey, 2005; Ríos-Serna et al., 2018). Variants of this gene include pathogenic, non-pathogenic, and others of uncertain significance. Pathogenic TBX5 variants promote the development of pathogenic changes and diseases through missense or non-sense substitutions, large deletions of multiple exons, frameshift mutations, and intragenic duplications (Spiridon et al., 2018). This pathology is conserved in vertebrates and exists in the mouse HOS and zebrafish heartstring model. Even a slight deviation in TBX5 expression can alter the severity of the disease phenotype, which partially explains why the same mutation can have different outcomes.

Hand deformities sometimes lead to a diagnosis of congenital heart disease; however, a genetic investigation is more appropriate to diagnose and prevent complications in subsequent generations. TBX5 variants, such as those with non-sense, splicing, and missense mutations, were detected in two previous studies of 78 HOS patients and their family members as well as three other families (Jhang et al., 2015; Vanlerberghe et al., 2019). TBX5 variants in eight individuals from four unrelated families were also correlated with familial dilated cardiomyopathy and atrial septal aneurysm along with tricuspid atresia and pulmonary stenosis, which represented a novel phenotype that may have other underlying mechanisms (Shankar et al., 2017; Patterson et al., 2020). This finding supports the diverse roles of TBX5 in cardiovascular development and function and confirms the importance of long-term cardiac surveillance for individuals affected by HOS (Patterson et al., 2020). In the current case, the patient inherited the base T (reference C) at POS114793240 from her mother and base C (reference T) at POS114793297 from her father, both of which belong to the 3′-UTR of the TBX5 gene. The two dbSNP IDs were benign based on the ClinVar analysis. HOS is caused by a mutation in TBX5, which has been identified in more than 70% of cases (Spiridon et al., 2018). The patient’s symptoms were not as severe as those of other HOS patients with mutations and SNPs at TBX5 exon sites. Therefore, we hypothesized that the two dbSNP IDs and their interactions with miRNAs may be the cause of her morbidity.

miR-98-5p regulated the myocardial differentiation of mesenchymal stem cells by targeting TBX5 in rat bone marrow samples, and miR-10a and miR-10b targeted the 3′-UTR of TBX5 to repress its expression in human embryonic kidney 293T cells (Wang et al., 2014; Sun et al., 2018). miR-182-5p exerted an evolutionarily conserved role as a TBX5 effector in the onset of cardiac propensity for arrhythmia in zebrafish, thereby mediating the relationship between TBX5, arrhythmia, and heart development (Guzzolino et al., 2020). A marked sensitivity in the developing heart of TBX5 gene-edited mice to Tbx5 dosage demonstrated the “rheostatic” control of Tbx5, which elucidates the disrupted transcriptional and cellular mechanisms in congenital heart defects (Mori et al., 2006). In this study, we conducted sequence alignment between the TBX5 3′-UTR regions, including rs883079 and rs10850326, and relevant miRNAs, including has-miR-98-5p, has-miR-10a, has-miR-10b, and has-miR-182-5p, and found that the binding of miRNAs and TBX5 3′-UTRs was promoted in HOS to further decrease TBX5 expression, thus inducing the transcription of HOS-promoting genes.

The mechanisms mediated by miRNAs and TBX5 may be spatiotemporally regulated, which may explain why the patient’s father and mother had SNPs and mutations but not HOS. The patient also inherited the SALL4 SNP in the exon area from both her father and mother. In zebrafish, SALL4 acts downstream of TBX5 and is required for pectoral fin outgrowth; mutations at the SALL4 locus on chromosome 20 resulted in a range of clinically overlapping phenotypes, including Okihiro syndrome, HOS, acro-renal-ocular syndrome, and thalidomide embryopathy (Harvey and Logan, 2006). Koshiba-Takeuchi et al. (2006) demonstrated that TBX5 regulated SALL4 expression in the developing mouse forelimb and heart, and mice heterozygous for the gene trap allele of SALL4 showed limb and heart defects that mirrored the human disease. TBX5 and SALL4 interact both positively and negatively to regulate the patterning and morphogenesis of the forelimb and heart. Thus, a positive and negative feed-forward circuit between TBX5 and SALL4 may potentially regulate the patterning of the embryonic limb and heart and represent a unifying mechanism for HOS. However, the exact consequences and mechanisms of the variants in SALL4 are unknown and require further research.

We identified 578 exons, along with 498 3′-UTRs and 346 5′-UTR mutations, in the patient using whole-exome sequencing, including the female urogenital genes PKD1, SON, and BMPR1B, as well as the reproduction-related genes TEK, COL11A1, ANKRD11, LEMD3, and SPTB, which was consistent with the findings of Tian et al. (2022).

This study had some limitations. To further explore the effects of TBX5 on cardiomyocytes, the HL-1 cell line was used with TBX5 knockdown. Quantitative polymerase chain reaction and chromatin immunoprecipitation analyses revealed that TBX5 binds to the TEKT2, TEKT4, and SPTB promoter regions to promote gene transcription. Further studies on mouse models and human cohorts are required. In addition, we only carried out sequence alignments between the TBX5 3′-UTR regions and previously reported miRNAs, and further validation is still needed. The effects of other genes on HOS formation and progression still need to be investigated.

We provide a novel gene signature of HOS, including TBX5 3′-UTR and downstream genes, such as SALL4 SNP, which may be a potential therapeutic target. Our findings support a novel TBX5-related pathogenic mechanism in HOS, though further experimental validation is needed. If this mechanism is correct, novel approaches can be devised for gene expression correction using pharmacological agents.

## Data Availability

The datasets for this article are not publicly available due to concerns regarding participant/patient anonymity. Requests to access the datasets should be directed to the corresponding author.

## Abbreviations

HOS, Holt–Oram syndrome; miRNA, microRNA; SNP, single-nucleotide polymorphism; UTR, untranslated region.
